# Household characteristics as predictors of access to paediatric malaria treatment in Homa-Bay County, Kenya

**DOI:** 10.1186/s13104-019-4514-7

**Published:** 2019-08-07

**Authors:** Maurice O. Kodhiambo, Beatrice K. Amugune, Julius O. Oyugi

**Affiliations:** 1 School of Pharmacy, Kenyatta University, P.O. Box 43844-00100, Nairobi, Kenya; 2 Department of Pharmaceutical Chemistry, University of Nairobi, Nairobi, Kenya; 3 Department of Medical Microbiology, University of Nairobi, Nairobi, Kenya

**Keywords:** Household, Access, Paediatric malaria, Homa-Bay

## Abstract

**Objective:**

To investigate the influence of socioeconomic household characteristics on access to paediatric malaria treatment in Homa Bay County, Kenya.

**Results:**

From univariate analysis, treatment with analgesics only in a community health center or a faith-based organization, self-employment, urban residence and residing in a sub-county other than Suba or Mbita showed significant association with access to paediatric antimalarial treatment. However, on multivariate analysis, urban residence, education, income of 10,000 to 30,000 and information from peers were the most statistically significant predictors of access to treatment. Urban households were 0.37 times more likely to access treatment than rural ones. Having primary, secondary or post-secondary education conferred 0.25, 0.14 and 0.28 higher chance of access to paediatric malaria treatment respectively compared to those with no formal education. Those with monthly income levels of 10,000 to 30,000 shillings had 0.32 higher chance of accessing treatment compared to those with less than 5000 shillings.

## Introduction

Children in the tropics bear the greatest burden of malaria. Household level access to antimalarial treatment for children with malaria varies greatly from one country to another. For example, a study in four African countries indicated access ranging from 3.6% for Ethiopia to 64.5% for Uganda. Those who accessed the artemisinin combination therapy (ACT) varied from 32.2% in Zambia and almost 100% in Tanzania [1]. Apart from knowledge, socio-economic factors such as having health insurance, ability to pay for care, positive attitude and perceived social support have also been associated with prompt access to malaria treatment [2]. Having medical insurance and distance traveled to health facility has generally been associated with health demand [3]. From a study in Burkina Faso, possession of antimalarial drugs at the households was associated with urban residence, level of education of household head, having young children, and high socio-economic status [4]. These findings thus show that differences in political and health policy characteristics between countries may be important in enabling households to access treatment. In Kenya, it is empirically documented that household size, household head, income source, amount of monthly income and age of the household head significantly influence access to care [5]. It is however not yet clear to what extent the new policy of health care service devolution in Kenya has impacted on access to malaria care. The study set out to investigate the association between household characteristics access to paediatric malaria treatment in Homa Bay County, Kenya after implementation of devolution of healthcare.

## Main text

### Study design and settings

The study was conducted as a population-based survey. Data was collected from household heads within their households between August 2016 and May 2017. The study was carried out in Homa Bay County which is located around the shores of Lake Victoria in Western Kenya. It is approximately 400 km South West of the Kenyan capital city, Nairobi.

### Study participants

The study population comprised all household heads in the eight sub-Counties of Homa-Bay County, Kenya. Data was collected from household heads as identified by the members of the household present during data collection. The decision to interview the household head only was arrived at baseline survey when we realized that most of the households in this rural County were headed by authoritarian males. Ignoring their power in household decisions would therefore have jeopardized our study as they would take it as insubordination. If the household head was absent, the household was skipped for the net until the sample size is attained.

### Sampling

Homa-Bay County was divided into blocks of eight sub-counties. Each block was further divided into urban and rural sub-blocks. For each of the sub-blocks, a sub-location was randomly sampled by way of cluster sampling. From each sub-location, a simple random sample of 20 households for rural and 30 households for urban clusters was taken. The sample size was therefore 16 clusters. All the households in each of the clusters sampled were included in the study. Ten percent of the sample size was added to cater for non response making the total sample size to be 440. Those that responded were 406, representing 92.3% response rate.

### Data collection tool and procedure

Research assistants arrived in a household, introduced themselves and requested the head of the household to either read the consent explanation or have it read to them before consenting. Upon granting the consent, the questionnaire was administered to them. Data collection was based on verbal autopsy and not on prescriptions or treatment records. This was because prescriptions and treatment records are often not available at the household level in Kenya. The questionnaire was designed to exclude culture sensitive questions such as household size and number of children in the household. In the culture of the rural Luo community, it is believed that if you count children, they will die soon in the order they are counted. We thought it was ethical to respect the culture of the respondents.

### Statistical analyses

Data was collected then cleaned, sorted and coded before entering on an excel spreadsheet followed by analysis by the R statistical software. General descriptive statistics analysis was performed prior to logistic regression to elicit the predictors of access to malaria treatment. Odds ratios and p-values we generated to assist in the analysis. The p-values were considered significant at the level of 0.05.

### Confounders

Potential confounders such as age and sex of household head as well as having medical insurance were recorded at the baseline interview prior to the survey.

## Results

### Access to paediatric malaria treatment at the household level

For those respondents that reported that their child had a malaria symptom within 1 month prior to the survey, several questions were posed to assess the level of access to malaria treatment. The results of the descriptive analysis of the responses are captured in Table 1.

**Table 1:** Access to paediatric malaria treatment by the households

Characteristics	Frequency	%
Child had malaria symptoms in the past month		
Yes	371	91.4
No	35	8.6
Severity of malaria symptoms when child was sick		
Not serious	86	21.2
Very serious	320	78.8
Medicines available at home		
None	216	53.2
ACT and analgesics	5	1.3
ACT only	78	19.2
Analgesic only	7	1.7
Other medicines	100	24.6
Drugs prescribed on visit		
ACT only	170	41.9
Antibiotic only	8	2.0
ACT + antibiotic	17	4.2
ACT + analgesic	43	10.6
ACT + other antimalarials	11	2.7
Non-ACT antimalarial	80	19.7
Analgesic only	31	7.6
Do not know	46	11.3
Was blood drawn for diagnosis of malaria?		
Yes	310	76.4
No	62	15.0
Not sure	34	8.6
If the diagnosis was done what was the result		
Positive	207	51.0
Negative	103	25.4
Can’t remember	96	23.6
Opinion on treatment cost		
Affordable	199	49.0
Not affordable	207	51.0
Availability of anti-malarial drugs		
Regularly available	166	40.9
Not regularly available	240	59.1

### Association between household characteristics and access to paediatric malaria treatment

The potential predictors of access were subjected to univariate analysis of how they individually regressed on household access to paediatric malaria treatment. A summary of the findings is as presented in Table 2.

**Table 2:** Univariate analysis of predictors of household access to paediatric malaria treatment

Characteristics	Access			
	Accessible	Inaccessible	OR (95% CI)	P-value
	n (%)	n (%)		
Drugs given				
Antibiotic only	6 (2.8)	2 (1.1)	1	
ACT + anti-biotics	8 (3.6)	9 (4.8)	3.38 (0.52–21.73)	0.189
ACT + analgesic	25 (11.5)	18 (9.6)	2.16 (0.39–11.95)	0.369
ACT + other anti-malarial	4 (1.8)	7 (3.7)	5.25 (0.69–39.48)	0.096
ACT only	118 (54.4)	52 (27.5)	1.32 (0.26–6.77)	0.737
Analgesic only	9 (4.1)	22 (11.6)	7.33 (1.24–43.41)	*0.017*
Non-ACT anti-malarial	21 (9.7)	59 (31.2)	8.43 (1.58–45.05)	*0.004*
Cannot remember	26 (12.0)	20 (10.5)	1.02 (0.53–1.34)	*0.560*
Was blood drawn for diagnosis?				
Yes	172 (78.2)	138 (74.2)	1	
No	27 (12.3)	35 (18.8)	1.62 (0.93–2.80)	0.085
Cannot remember	21 (9.5)	13 (7.0)	1.55 (0.87–2.41)	0.096
Where did you seek care				
Government hospital	128 (61.0)	86 (41.7)	1	
Private hospital	19 (9.0)	20 (9.7)	1.57 (0.79–3.11)	0.197
Pharmacy	17 (8.1)	16 (7.8)	1.40 (0.67–2.92)	0.368
Community Health Centre	2 (1.0)	7 (3.3)	5.21 (1.06–25.68)	*0.025*
Shop	17 (8.1)	11 (5.3)	0.96 (0.43–2.16)	0.927
Traditional practitioner	8 (3.7)	11 (5.3)	2.05 (0.79–5.29)	0.133
Other	2 (1.0)	8 (3.9)	5.95 (1.23–28.71)	0.126
Faith Based Org. (FBO)	6 (2.9)	13 (6.3)	3.22 (1.18–8.81)	*0.017*
Not sure	11 (5.2)	24 (11.7)	4.31 (0.82–6.34)	0.71
Occupation				
Business	15 (6.8)	21 (11.2)	1	
Farmer	32 (14.6)	42 (22.5)	0.94 (0.42–2.10)	0.875
Salaried employment	27 (12.3)	25 (13.4)	0.66 (0.28–1.56)	0.344
Self employed	125 (57.1)	85 (45.4)	0.49 (0.24–1.00)	*0.046*
No response	20 (9.2)	14 (7.5)	0.73	0.512
Household status				
Rural	55 (26.6)	86 (42.3)	1	
Urban	144 (69.6)	87 (43.7)	0.39 (0.25–0.59)	*< 0.001*
No response	8 (3.8)	26 (13.0)	0.93(0.62–1.26)	0.077
Sub-County				
Homabay	14 (6.5)	33 (17.5)	1	
Kabondo	26 (12.0)	27 (14.3)	0.44 (0.19–1.01)	*0.049*
Kasipul	42 (19.4)	6 (3.2)	0.06 (0.02–0.17)	*< 0.001*
Mbita	7 (3.2)	44 (23.3)	2.67 (0.96–7.35)	0.053
Ndhiwa	36 (16.6)	9 (4.7)	0.11 (0.04–0.28)	*< 0.001*
Rangwe	30 (13.8)	10 (5.3)	0.14 (0.47–2.79)	*< 0.001*
Suba	13 (6.0)	35 (18.5)	1.14 (0.47–2.79)	0.770
Rachuonyo	31 (14.2)	9 (4.8)	0.12 (0.05–0.33)	*< 0.001*
No response	18 (8.3)	16 (8.4)	1.54 (0.79–1.55)	0.063

From the univariate analysis, the predictors that showed statistically significant association with access to paediatric malaria treatment were seeking care from a community health center OR = 5.21 (1.06–25.68) (p = 0.025), and from a faith-based organization (OR = 3.22 (1.18–8.81) (p = 0.017). Other factors that were statistically significant were self-employment OR = 0.49 (0.24–1.00) (p-value = 0.046), living in an urban area of Homa-Bay County OR = 0.39 (0.25–0.59) (p < 0.001), and being a resident of Kabondo OR = 0.44 (0.19–1.01) (p = 0.049), Kasipul OR = 0.06 (0.02–0.17) (p < 0.001), Ndhiwa OR = 0.11 (0.04–0.28) (p ≤ 0.001), Rangwe OR = 0.14 (0.47–2.79) (p ≤ 0.001) or Rachuonyo OR = 0.12 (0.04–0.28) (p ≤ 0.001) sub-counties.

All the predictors were then subjected to multiple regression analysis using the R-Studio software. Results of the multivariate logistic regression analysis are shown in Table 3.

**Table 3:** Multiple logistic regression analysis of predictors of Household access to paediatric malaria treatment

Predictor	Coeff.	OR	95% CI	p-value
Place of residence				
Urban	0.997	0.37	(− 1.516, − 4.780)	*< 0.001*
Education				
Primary	1.396	0.25	(− 2.411, − 0.380)	*0.007*
Secondary	1.969	0.14	(− 3.001, − 0.932)	*< 0.001*
Post-secondary	1.291	0.28	(− 2.466, − 0.114)	*0.032*
Occupation				
Farming	0.609	0.54	(− 1.643, 0.424)	0.248
Salaried employment	0.300	0.74	(− 1.312, 0.713)	0.562
Self-employment	0.608	0.54	(− 1.499, 0.284)	0.181
Monthly income (Kshs)				
5000–10,000	0.197	0.82	(− 1.771, 0.380)	0.502
10,000–3,0000	1.147	0.32	(− 2.139, − 1.554)	*0.023*
30,000–50,000	0.584	1.79	(− 0.478, 1.647)	0.281
> 50,000	0.515	1.67	(− 1.813, − 0.843)	0.447
Source of information on malaria				
Peers	2.930	18.736	(0.825, 5.036)	*0.006*
Hospital	1.625	5.079	(− 0.102, 3.353)	0.065
Media	1.385	4.000	(− 0.325, − 3.096)	0.112
Pharmacy	1.603	4.968	(− 0.383, − 3.589)	0.114
Public health officers	0.667	1.948	(− 1.191, − 2.525)	0.482
Time to care seeking				
Delayed	− 0.351	0.704	(− 0.848, 0.146)	0.116

On multivariate analysis, urban residence, education at all levels, monthly household income of 10,000 to 30,000 and information from friends stood out as the most statistically significant predictors of access to paediatric malaria treatment in Homa-Bay County.

## Discussion

The prevalence of malaria among children with fever one month prior to the study was 66.8%. This percentage is much higher than that estimated in a study in Maiduguri city [6]. This is possibly due to incorrect or inconsistent use of the bed nets. Even if they used them consistently, it was also evident that they were treated with mosquito repellants only intermittently. However, there is also a possibility of over diagnosis which has been shown to be common in malaria endemic areas [7]. Government owned health care facilities were most preferred as the first place for seeking care. Time lapse between onset of symptoms and seeking treatment was significantly associated with education and occupation of the head of the household, severity of illness, having medicines at the household, income and residence. Similar findings were reported by Kassile et al. [8] and Angwin et al. [9]. They stated that delays in care seeking for children with fever was determined by communal beliefs, socio-economic status, education and occupation of household head. Tipke et al. [4] also found out that having drugs at the household usually delays care seeking. In Africa, most caregivers prefer to manage childhood fevers at home or to seek folklore medicines before going for conventional treatment [2, 3, 10]. Perhaps they do so because as much as they may be physically able to access care, financial barriers do exist. There are three universally accepted dimensions of access to health care namely availability, acceptability and affordability. In this study, we operationally defined access as both physical and financial ability to utilize health care. From univariate regression, access to malaria care was associated with medicine prescribed, seeking initial care from a community health centre or FBO, self employment, urban residence and sub-county. However, on multivariate analysis, access was found to be statistically significantly associated only with education, income, residing in an urban neighbourhood and receiving information about malaria from peers. Researchers in Rwanda reported similar findings that access to malaria treatment is associated with knowledge and socio-economic status of the household head as well as social support [2]. We considered receiving information on malaria from peers as an indicator of having social support. Perceptions about treatment outcomes were associated with visiting a government health facility, urban residence and being self-employed. These findings concur with those of a study in Tanzania which reported that people who were financially more stable preferred government health facilities as they were associated with better outcomes [11]. Similar findings were recorded from a Burkinabe [12] where treatment outcomes were associated with residence, education and socio-economic status of the household head, delay in initiating treatment and home based care. Some participants reported to have been given only ACT. This was probably because they informed the clinician that they had the other medicines at home. Alternatively there could have been drug stock outs for the other medicines, which is a frequent phenomenon in rural Kenya even for the most essential of drugs.

## Conclusion

The findings of this study provide sufficient scientific evidence that socioeconomic status and level of education of the household head, rural or urban residence and perceived social support are key predictors of household access to paediatric malaria treatment in Homa Bay County. Further research should probe on how these factors affect the overall health systems performance.

## Limitations

This study was conducted as a cross sectional survey thus making it difficult to ascertain a causal relationship between the exposure and outcome. Only associations may be inferred. Additionally, since all data was collected by way of respondent reports, there is a possibility of recall bias.

## Data Availability

All the necessary data are presented herewith. However if needed, raw data on excel format can be availed on reasonable request from the corresponding author.

## Abbreviation

ACT: artemisinin combination therapy
